# Homologous metal heteromaterials for ultrastable eletrocatalysis

**DOI:** 10.1093/nsr/nwaf411

**Published:** 2025-09-24

**Authors:** Wei Li, Dongyuan Zhao

**Affiliations:** Department of Chemistry, Laboratory of Advanced Materials, Shanghai Key Laboratory of Molecular Catalysis and Innovative Materials, and State Key Laboratory of Porous Materials for Separation and Conversion, Fudan University, China; Department of Chemistry, Laboratory of Advanced Materials, Shanghai Key Laboratory of Molecular Catalysis and Innovative Materials, and State Key Laboratory of Porous Materials for Separation and Conversion, Fudan University, China

Controlling interfacial bonding types such as van der Waals interaction and chemical bonding is crucial for developing heteromaterial structures and functions. Taking electrochemical gas evolution reactions as an example, extreme high-current-density (HCD) working conditions often impose demands on the mechanical and electrical properties of heteromaterial interfaces [1–3]. Conventional heterostructure catalysts have small van der Waals interaction energy (0.1–1 J m^−2^) at the interface (e.g. commercial Pt/C catalyst), which is insufficient to withstand high bubble adhesion energy (1–100 J m^−2^), resulting in the catalyst detaching and stability decay under HCD conditions [4,5]. Furthermore, interfacial defects and band structure misalignment in heterostructure catalysts slow down the charge-transfer kinetics, thus diminishing catalytic activity [6–8]. Meeting the two criteria of robust interfacial binding and low interfacial electrical resistance is essential yet challenging for heterostructure catalysts, particularly under industrial HCD operation conditions.

**Figure 1.**
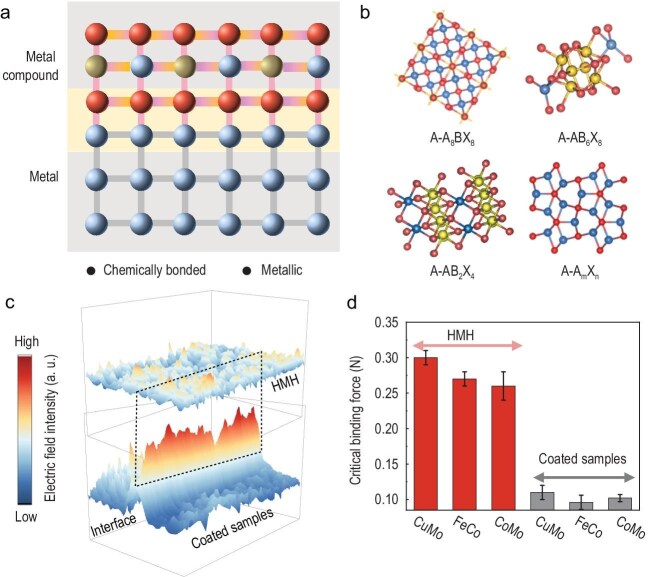
Family of HMHs. (a) Schematic of HMHs with a chemically bonded metallic interface. (b) Atomic models of four types of HMHs. The A metal includes Fe, Co, Ni or Cu, the B metal includes Fe, Ni or Mo and the X represents S or Se chalcogens. (c) Three-dimensional mappings of interfacial electric field intensity of CuMo-HMH and CuMo-coated samples. (d) Comparison of critical binding forces of HMHs and coated samples.

Recently, the research group led by Bilu Liu of Tsinghua University prepared a family of homologous metal heteromaterials (HMHs) featuring a chemically bonded metallic interface for electrocatalysis, addressing the problems of catalyst instability and high charge-transfer resistance [9]. The HMHs were synthesized by metal source diffusion into precursors to obtain four categories and 20 materials. The HMHs feature a chemically bonded metallic interface between metal and metal compounds, eliminating the charge-transfer barrier and forming

a strong mechanical binding force (Fig. 1a and b). In detail, uniform electric field distribution at the interface of HMHs confirms that the metallic interface in HMHs has a negligible electric field intensity difference across the interface, while there is a big difference in conventional coated samples (Fig. 1c). The robust interfacial chemical bonds of HMHs make the critical binding force of HMHs two to three times larger than those of coated samples, effectively preventing catalyst detachment from the substrate under HCD operation (Fig. 1d).

The authors further used an *in situ* optical polarization imaging technique for high-throughput screening of HMH catalysts. Owing to their robust interfacial mechanical stability and unimpeded interfacial charge transfer, the screened HMH catalysts exhibit optimal hydrogen evolution reaction and oxygen evolution reaction performance at 2 A cm⁻², with overpotentials of 336 and 349 mV, respectively. Remarkably, HMH catalysts demonstrate exceptional long-term stability in an anion exchange membrane water electrolyser, exhibiting a voltage decay rate of 1.06 μV h⁻¹ at 500 mA cm⁻² over 1000 hours. This decay rate represents one of the lowest values at HCD reported to date, demonstrating that HMH catalysts have the highest stability.

This study represents a major breakthrough in the preparation of chemically bonded metallic interfaces in metal compound heterostructures. The work advances heterointerface construction and the elucidation of novel interfacial conductance mechanisms, and offers potential solutions for electrical contact issues in electronics and energy devices. In future work, a closed-loop artificial intelligence laboratory can be established to unify experimentation, simulation and machine-learning algorithms, further accelerating functional interface design and material screening. Additionally, the scalable and cheap production of HMHs should be done to promote industrial uses of such materials.
